# Huge polypoid endometriosis: report of a case and review of the literature

**DOI:** 10.1007/s13691-015-0220-z

**Published:** 2015-04-29

**Authors:** Akiko Yamamoto, Tomoka Usami, Eiji Kondo, Kazuyoshi Kato, Teiichi Motoyama

**Affiliations:** 1 Department of Gynecology, Cancer Institute Hospital, Ariake 3-8-31, Koto, Tokyo 135-8550 Japan; 2 Department of Pathology, Cancer Institute Hospital, Ariake 3-8-31, Koto, Tokyo 135-8550 Japan

**Keywords:** Polypoid endometriosis, Decidual change, Uterine serosa, Adenosarcoma

## Abstract

‘Polypoid endometriosis’ is a rare variant of endometriosis. We describe a case of an extremely large polypoid endometriosis mimicking a malignant tumor. A 37-year-old nulliparous woman was referred due to the rapid growth of an endometriotic cyst of the ovary and a high serum CA125 level. MRI revealed solid components in the pelvic mass. These preoperative clinical data were compatible with an ovarian carcinoma. A frozen section of the tumor biopsy showed as if an adenosarcoma, but finally the diagnosis of polypoid endometriosis with decidual change was made on permanent section. Polypoid endometriosis is a part of the differential diagnosis for malignant tumors in women with endometriosis, and we should consider carefully decision making for treatment.

## Introduction

Endometriosis is a relatively common disease among women of childbearing age. However, ‘polypoid endometriosis’ is a considerably rare form of endometriosis that may mimic a neoplasm on clinical, intraoperative, and gross examination [1].

Here, we report a case of huge polypoid endometriosis that grew rapidly occupying the whole pelvis and made it difficult to make a proper diagnosis.

## Clinical summary

**Fig. 1:**
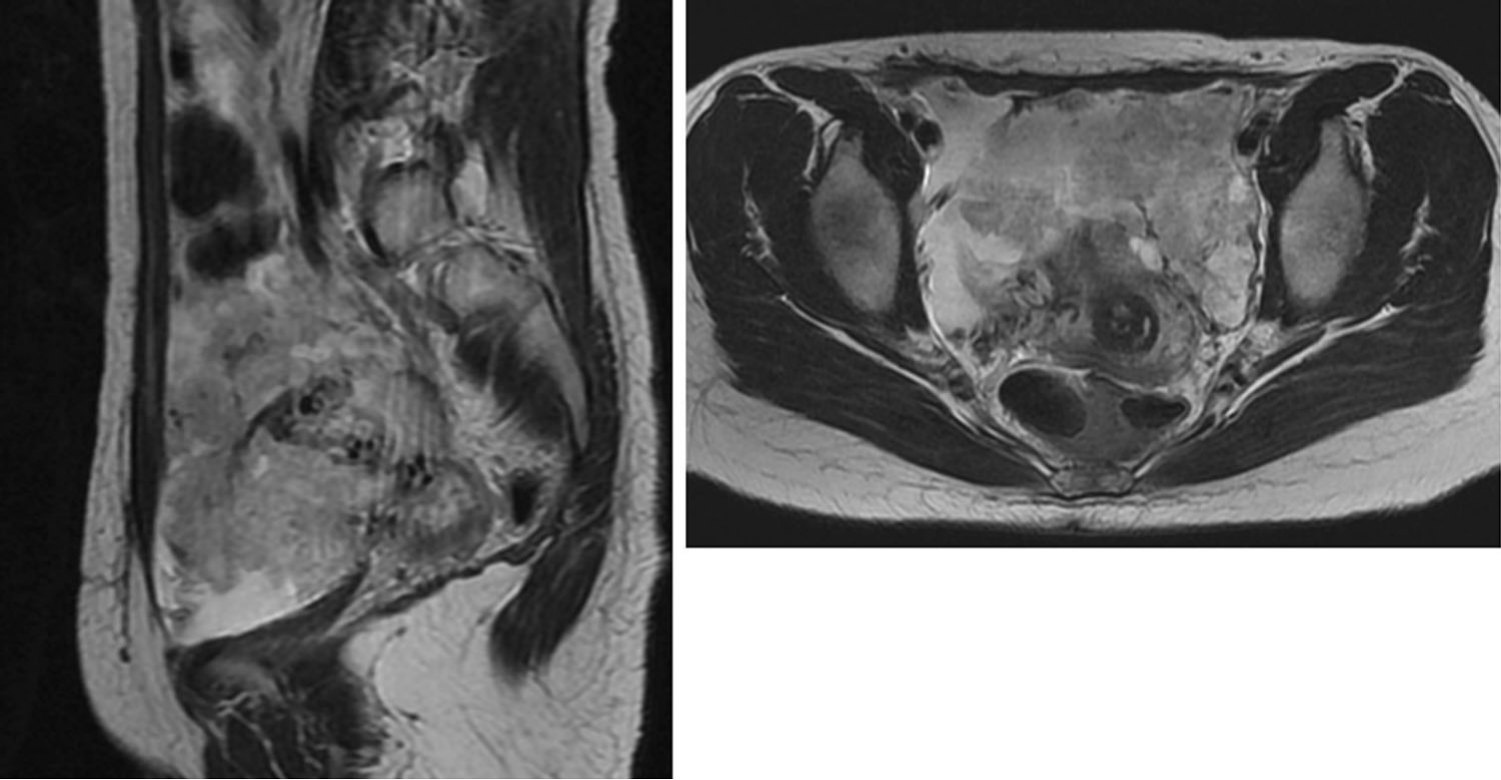
Pelvic MRI: T2-weighted imaging shows irregular shaped mass with *solid* components occupying the whole pelvis

A 37-year-old nulliparous woman was referred due to a rapid growth of an endometriotic cyst of the ovary and a high serum CA125 level. The ovarian mass had grown from 3 to over 20 cm in 6 months, and the serum CA125 level elevated to 3263 U/ml (normal <35 U/ml). She had a long history of various hormonal therapies and repeated laparotomy for severe endometriosis. She had no history of treatment with Tamoxifen. Magnetic resonance imaging (MRI) revealed solid components in the mass to have high signal intensity on T2-weighted imaging (Fig. 1) and irregular thickness of the omentum and the peritoneum. These clinical findings made us consider a malignant ovarian tumor, especially a papillary serous adenocarcinoma with peritonitis carcinomatosa. A surgical abdominal exploration revealed a huge polypoid mass occupying most of the pelvis, and a frozen section suggested an adenosarcoma. However, the permanent sections of surgically resected mass disclosed that the lesion was polypoid endometriosis.

## Macroscopic findings

**Fig. 2:**
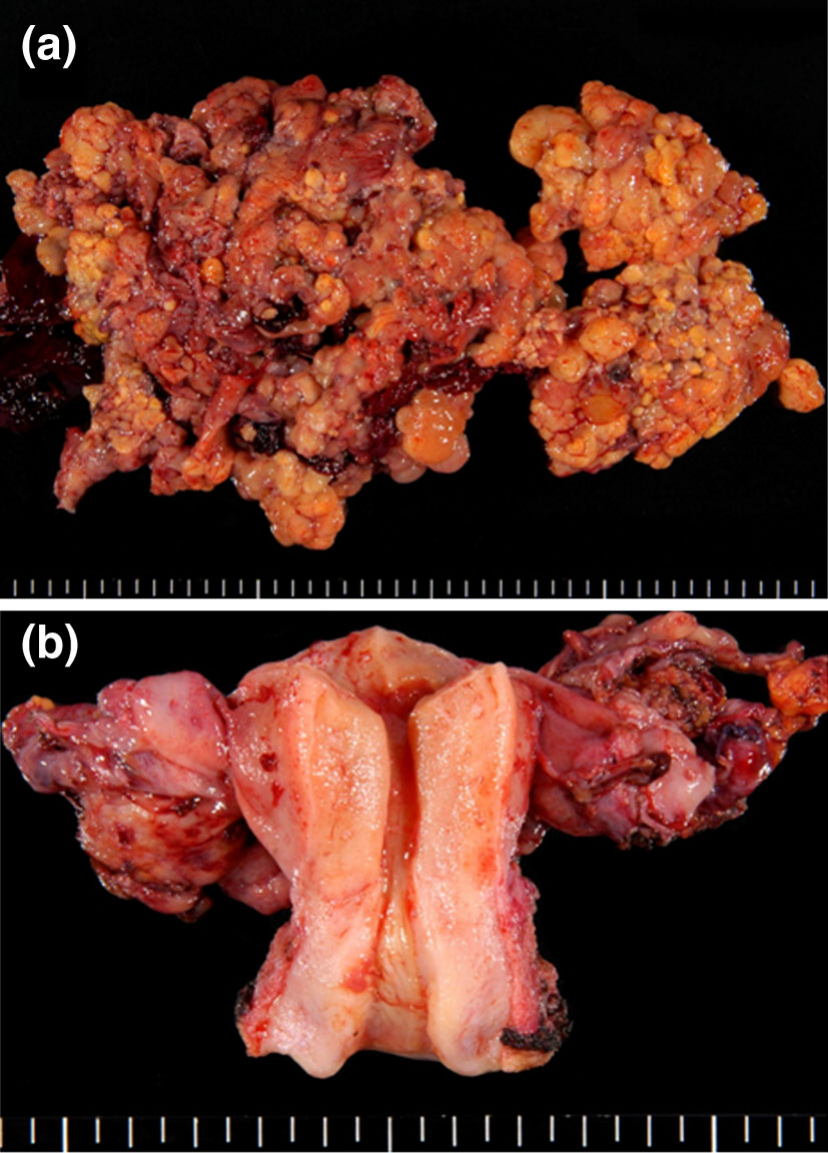
Macroscopic findings: **a** cauliflower-like polypoid mass arising from the serosa of the uterus. **b** Both ovaries are present separately from the tumor

The cauliflower-like polypoid mass arose from the serosa of the uterus and was whitish yellow in color and 20 cm (Fig. 2a). Both ovaries were cystic with chocolate-colored fluid (Fig. 2b). Whitish small foci were also found in the omentum and the peritoneum.

## Microscopic findings

**Fig. 3:**
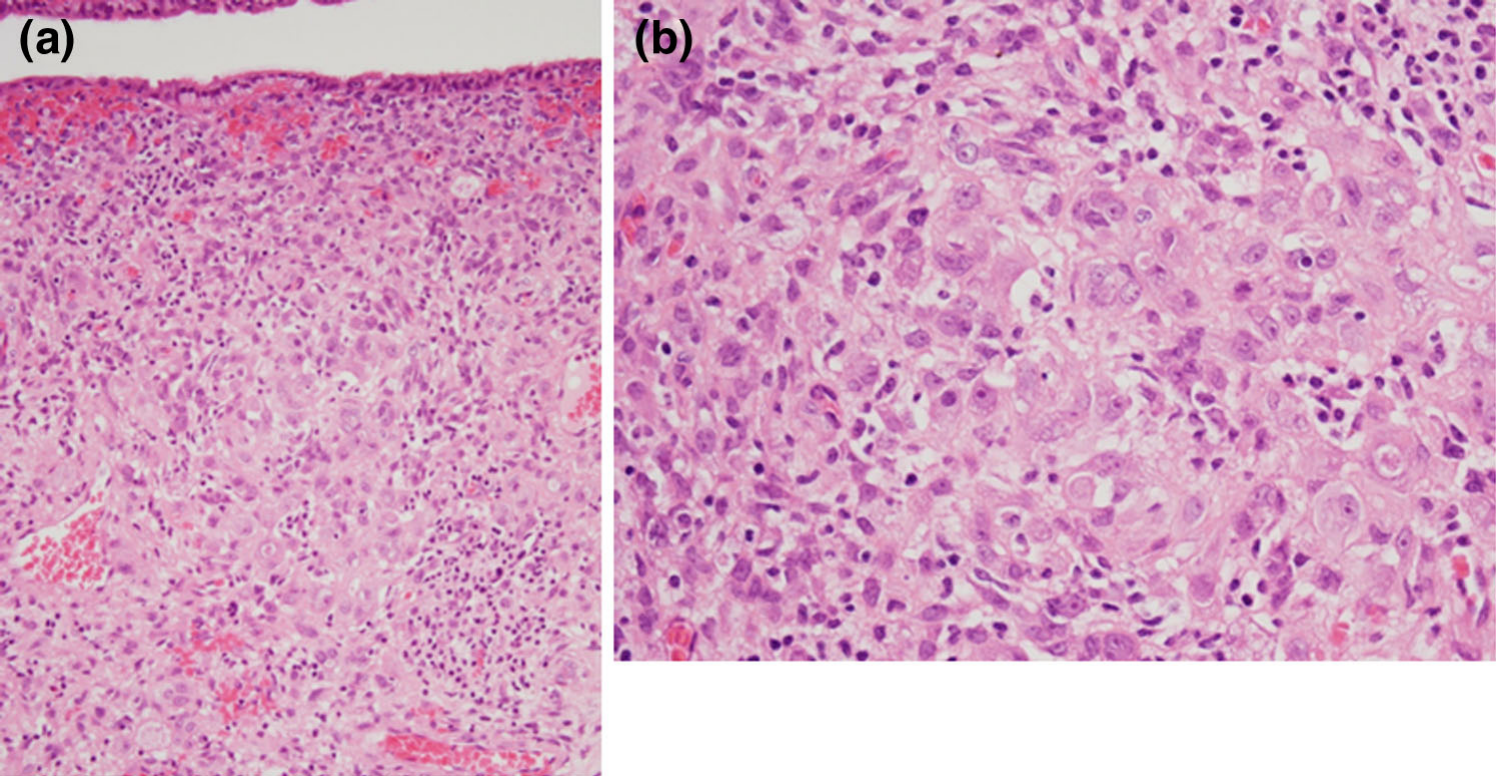
Microscopic findings: the polypoid lesion consisted of endometriosis with decidual change. **a** The tumor is covered with monolayered columnar epithelia on the surface. A dense cellular layer and relatively loose cellular layer were located under the epithelial layer. **b** Stromal cells are polygonal having abundant cytoplasm and an enlarged nucleus with prominent nucleoli

**Fig. 4:**
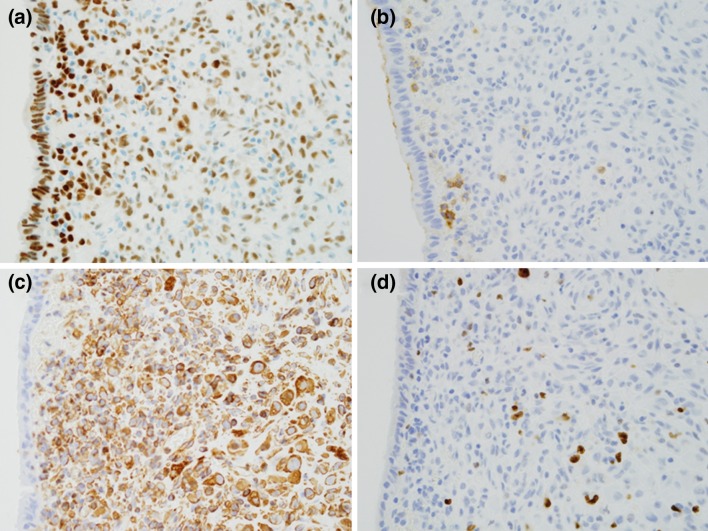
Immunochemical findings: **a** estrogen receptor is positive on most epithelial and stromal cells, **b** a few stromal cells are positive for CD10, **c** most stromal cells are strongly positive for vimentin, **d** MIB-1 index is 5 %

**Fig. 5:**
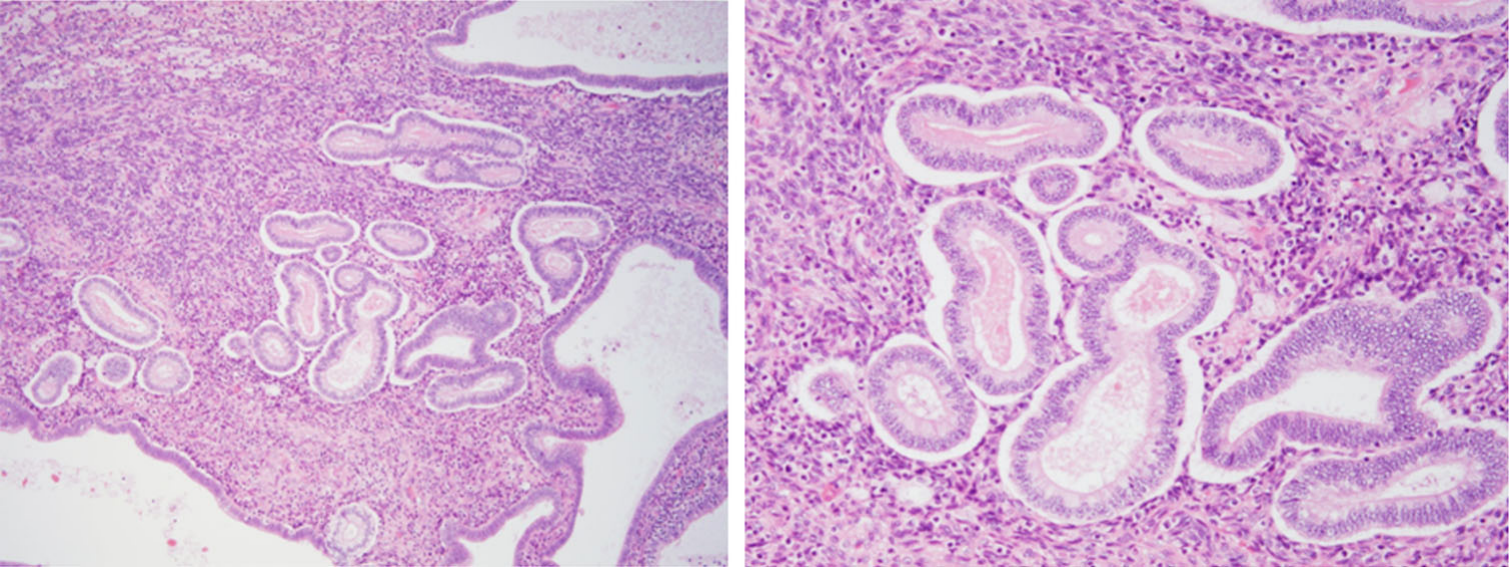
Some parts of the tumor are composed of endometrium-like tissue showing the secretory phase

The polypoid lesion was covered with monolayered columnar to cuboidal epithelial cells on the surface and had a dense cellular stroma under the epithelial layer (Fig. 3a). The polypoid lesion of more than half of the tissue was occupied by stroma. This dense cellular stroma mainly consisted of polygonal cells having abundant cytoplasm and enlarged nucleus with prominent nucleoli (Fig. 3b). Such cells were positive for vimentin and estrogen receptor. A few cells were positive for CD10. MIB-1 index was 5 % (Fig. 4a–d). We found that this stroma, extended one-third of whole stroma roughly, showed decidual reaction. The internal part of polypoid lesion was composed of an admixture of endometriotic glands and stroma. There were some areas composed of endometrium-like tissue showing the secretory phase (Fig. 5). The typical endometriosis was seen in both ovaries, serosa of the uterus, omentum, and peritoneum. Based on these microscopic findings, the diagnosis of polypoid endometriosis with frequent decidual change was made.

## Discussion

The term ’polypoid endometriosis’ was first used by Mostoufizadeh and Scully [1] in 1980. They described polypoid endometriosis as “endometriotic tissue with histologic features simulating those of an endometrial polyp, and rarely, polypoid masses of endometriosis not only simulate malignant tumors at operation but may also recur after operative removal.”

Because of the rarity, there was only one large series on such cases, in which Parker et al. [6] performed 24 cases of clinicopathologic analysis. According to their study, the mean age of the patients was 52.5 years: 60 % were older than 50 years, that is to say that this variant of endometriosis emerged among the elder generation compared with the age range of 25–30 years in the conventional type [7]. Occasionally, polypoid endometriosis was involved in multiple sites and the most common sites were the large bowel and the ovary. Regarding the gross findings, the maximal size of lesions ranged from 0.4 to 14 cm, appeared as polypoid masses and had a cystic part in half of the cases [6]. The cause of polypoid endometriosis is still not clear; however, some cases may be ascribed to estrogenic hormonal stimulation. Parker et al. [6] stated that eleven women in 24 had been taking exogenous estrogen hormones after menopause. Meanwhile, Schlesinger et al. [8] mentioned a correlation between polypoid endometriosis and tamoxifen. Tamoxifen, which is widely used as an adjuvant therapy for breast cancer, exhibits an anti-estrogen effect on the breast while being an agonist on the uterus. Several cases [9, 10] of polypoid endometriosis in tamoxifen users can support that hyperestrinism is one of the causes of this disease. Furthermore, Othman et al. [11] reported a case of polypoid endometriosis following gonadotrophin-releasing hormone (GnRH) agonist. They postulated that the lesion developed as a rebound phenomenon upon withdrawal of GnRH agonist-induced hormonal suppression [12].

In view of pathological characteristics, Parker et al. defined polypoid endometriosis as “an admixture of benign, but sometimes atypical, appearing endometriotic glands and benign-appearing endometriotic stroma.” With regard to the stroma, stromal cell atypia was not observed in any case and in most cases appeared in the proliferative phase stroma. Focal decidual change and increasing stromal cellularity were also noted in some cases [6]. The main differential diagnosis should be an adenosarcoma. Microscopic characteristics of adenosarcoma are Müllerian-type glands without atypia in the malignant stromal component, periglandular cuffs of cellular stroma, and variable mitotic count [13, 14].

In the present case, the patient had been regularly checked for endometriosis since she was young. She had received laparotomy twice, and then in each resected tissue endometriosis without any evidence of malignancy was pathologically proved. While she had received several surgical treatments, she had also a variety of hormonal therapies such as oral contraceptives, GnRH agonist, and progestins rotationally. Administration of the oral progestins had been interrupted 6 months prior to her appearance at our hospital. Through her regular follow-up in these 6 months, the pelvic mass, considered to be an endometriotic cyst of the ovary at this point, grew from 3 to 20 cm, changed its shape with solid components, and the serum CA125 level rose gradually. These clinical findings convinced us of the presence of malignancy.

Compared to literature cases [1–12] (Table 1), our case was younger, considerably huge in size, and the lesions developed in the pelvis rapidly as soon as there was a break of hormonal therapy. The cause of this rapid growth of lesions is uncertain, but interruption of hormonal treatment could have contributed to hyperestrinism and raised polypoid masses in this case. The main mass was covered with epithelial cells without atypia, and stromal cells were mildly dense with enlarged nucleus with prominent nucleoli. Such findings led to a misdiagnosis of Müllerian adenosarcoma from frozen sections. We finally concluded that the mass was a polypoid endometriosis with decidual change since stromal atypia was mild and the MIB-1 index was 5 % on permanent sections. Probably, decidual change was caused by the prior progestin therapy or spontaneous ovulation in this case. We assume that decidualized stromal cells appeared as if a sarcomatous component.

**Table 1:** Polypoid endometriosis: review of the literature

References	Age (years)	Location	Hormonal status	Size of the biggest mass (cm)
Mostoufizadeh and Scully [1]	65	Both ovaries, pelvic tissue	None	NA
	47	Vagina	None	12
	55	Cervical os	ERT	NA
Chang and Natarajan [3]	58	Endometrium	ERT	2
Kaushal et al. [4]	27	Vagina	None	3.5
Lambrechts et al. [5]	29	Bladder	During pregnancy	3
Parker et al. [6].	55	Ureter and vagina	ERT	NA
	63	Adnexa	None	4
	64	Uterine serosa	None	10
	43	Peritoneum	ERT	13
	34	Retroperitoneum	ERT	8
	67	Colonic mesentery	None	1.2
	57	Cervix and vagina	None	14
	60	Ovary	EPRT	0.9
	78	Pelvic endometriosis	None	NA
	74	Omentum	ERT	5
	43	Ovary	None	10
	59	Ovary	None	NA
	40	Colonic mucosa	None	7
	NA	Colonic mucosa	None	3
	39	Peritoneum	None	8
	53	Colonic wall and peritoneum	ERT	7
	23	Pelvic endometriosis	None	12
	62	Ovary	ERT	7.2
	55	Uterine serosa	None	4.5
	52	Ovary	ERT	7
	56	Ureter	None	1.6
	40	Vagina	EPRT	3
	45	Vagina	None	2
	46	Colonic mucosa	None	4
Schlesinger and Silverberg [8]	65	Cervix and ovary	Tamoxifen	NA
Kraft and Hughes et al. [9]	47	Ovary	Tamoxifen	NA
Chang et al. [10]	Premenopause	Pelvic endometriosis	Tamoxifen	6
Othman et al. [11]	26	Endometrium	GnRH agonist	NA
Marugami et al. [12]	45	Ureter	GnRH agonist	
Current case	37	Uterine serosa	Various	20

*NA* not available, *EPRT* estrogen progestin replacement therapy, *ERT* estrogen replacement therapy

In conclusion, polypoid endometriosis is a part of the differential diagnosis for malignant tumors in women with endometriosis. We should be aware of the difficulty of making proper diagnosis pre- and intraoperatively and consider carefully decision making for treatment, especially for young women.

## References

[1] Mostoufizadeh M, Scully RE (1980). Malignant tumors arising in endometriosis. Clin Obstet Gynecol.

[2] Cantor JO, Fenoglio CM, Richart RM (1979). A case of extensive abdominal endometriosis. Am J Obstet Gynecol.

[3] Chang A, Natarajan S (2001). Polypoid endometriosis. Arch Pathol Lab Med.

[4] Kaushal S, Dadhwal V, Mathur SR (2010). Multifocal polypoid endometriosis in a young woman simulating vaginal and pelvic neoplasm. J Clin Pathol.

[5] Lambrechts S, Calsteren KV, Capoen A (2011). Polypoid endometriosis of the bladder during pregnancy mimicking urachal carcinoma. Ultrasound Obstet Gynecol.

[6] Parker RL, Dadmanesh F, Young RH (2004). Polypoid endometriosis: a clinicopathologic analysis of 24 cases and a review of the literature. Am J Surg Pathol.

[7] Eskenazi B, Warner ML (1997). Epidemiology of endometriosis. Obstet Gynecol Clin North Am.

[8] Schlesinger C, Silverberg SG (1999). Tamoxifen-associated polyps (basalomas) arising in multiple endometriotic foci: a case report and review of the literature. Gynecol Oncol.

[9] Kraft JK, Hughes T (2006). Polypoid endometriosis and other benign gynaecological complications associated with tamoxifen therapy—a case to illustrate features on magnetic resonance imaging. Clin Radiol.

[10] Chang CK, Chen P, Leu FJ (2003). Florid polypoid endometriosis exacerbated by tamoxifen therapy in breast cancer. Obstet Gynecol.

[11] Othman NH, Othman MS, Ismail AN (1996). Multiple polypoid endometriosis—a rare complication following withdrawal of gonadotrophin releasing hormone (GnRH) agonist for severe endometriosis: a case report. Aust NZ J Obstet Gynaecol.

[12] Marugami N, Hirohashi S, Kitano S (2008). Polypoid endometriosis of the ureter mimicking fibroepithelial polyps. Radiat Med.

[13] Eichhorn JH, Young RH, Clement PB (2002). Mesodermal (müllerian) adenosarcoma of the ovary: a clinicopathologic analysis of 40 cases and a review of the literature. Am J Surg Pathol.

[14] Gallardo A, Prat J (2009). Müllerian adenosarcoma: a clinicopathologic and immunohistochemical study of 55 cases. Challenging the existence of adenofibroma. Am J Surg Pathol.

